# Identification of a self-regulatory pheromone system that controls nymph aggregation behavior of rice spittlebug *Callitettix versicolor*

**DOI:** 10.1186/s12983-015-0102-4

**Published:** 2015-05-17

**Authors:** Xu Chen, Ai-Ping Liang

**Affiliations:** Key Laboratory of Zoological Systematics and Evolution, Institute of Zoology, Chinese Academy of Sciences, Beijing, 100101 China

**Keywords:** Spittlebug, Aggregation pheromones, Aggregation behavior, *n*-alkanes, *Callitettix versicolor*

## Abstract

**Background:**

Nymphs of many spittlebug species are known to aggregate in one spittle mass, a behavior which greatly benefits the survival of the developing nymphs. Little is known, however, about the precise mechanisms that induce and regulate aggregation. Here, we investigated the aggregation behavior of nymphs of the rice spittlebug *Callitettix versicolor*, and analyzed the chemical composition of spittle masses.

**Results:**

We identified six n-alkane compounds, namely un-, do-, tri-, tetra-, penta- and hexadecane in the spittle mass. Importantly, we showed that solitary spittle mass (SSM) and aggregation spittle mass (ASM) differed significantly in the amounts and composition of these compounds. While un-, do-, tri-, tetra-and hexadecane were overrepresented in SSM, pentadecane was found at significantly higher levels in ASM. Electrophysiological experiments showed that antennae responses to these six compounds were significantly higher than to both the hexane and the docosane control, which suggests a specific role of the six volatile alkanes as pheromones. In agreement with this hypothesis, behavioral tests revealed that five of the six compounds (e.g. un-, do-, tri-, tetra-, and hexadecane) acted as attractants across a wide concentration range. Thus, these five compounds allow recruitment of additional nymphs to a growing spittle mass. The sixth compound, pentadecane, attracted nymphs at low doses, whereas at higher doses, this effect vanished, suggesting that this alkane functioned as a repellent, thus preventing recruitment of additional individuals to a full aggregation in a spittle mass.

**Conclusions:**

In summary, our study identified a simple, yet fully functional feedback mechanism which allows aggregation at low nymph numbers, while preventing over-crowding beyond a set number of nymphs within one spittle mass. In conclusion, our study provides new insights into *C. versicolor* development and behavior that should greatly facilitate the identification of new approaches for pheromonal control of this pest.

## Introduction

Aggregation is a common phenomenon observed for various animal groups, for which it affects many spatial and temporal processes in the respective ecological systems [1]. Classically, aggregation has been viewed as an evolutionarily advantageous state, in which members derive benefits [2], such as protection and food exploration. Aggregation in the immature stages of insects has been documented in various taxonomic groups, and this phenomenon may occur passively through clumped egg-laying or actively through crowding. The latter is thought to be driven by aggregation pheromones [3]. Aggregated individuals obtain more benefits than when solitary, including more efficient resource use, overcoming of host resistance, and protection from both natural enemies and environmental conditions. On the other hand, individuals within aggregations experience higher costs than they would if they were solitary with competition for food and space may be the most severe challenge [1]. Hence, the aggregation size must be regulated to balance the benefits and costs of aggregation. Aggregation pheromones usually play an important role in this process.

**Figure 1:**
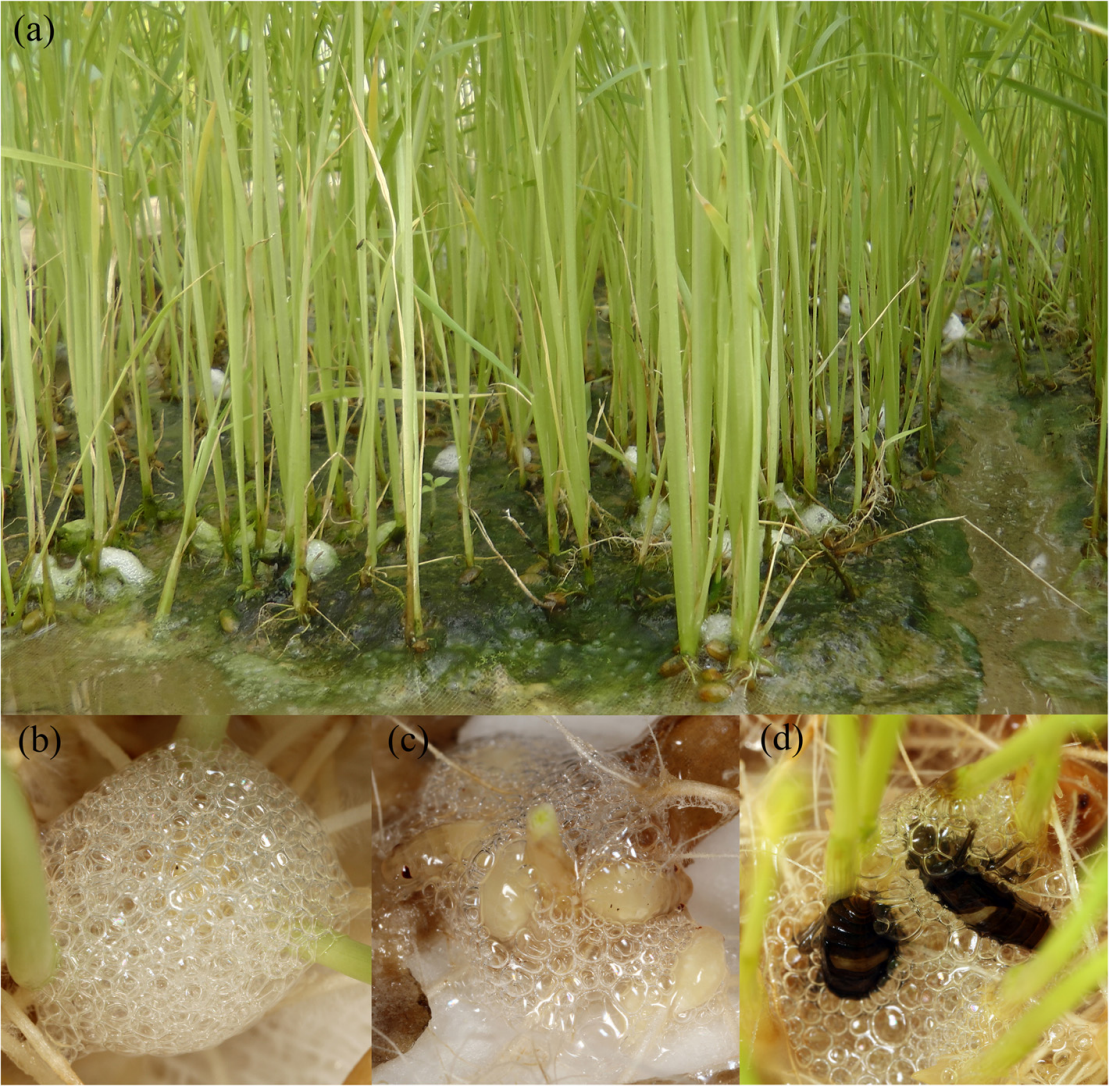
The spittle masses and aggregation behavior of *C. versicolor* nymphs. **(a)** The nymphs covered themselves with spittle masses at the roots of rice; **(b)** The spittle masses produced by nymphs; **(c)** The aggregation of second and third instar nymphs in one spittle mass; **(d)** The aggregation of two fifth instar nymphs in one spittle mass.

Spittlebugs (Hemiptera: Cercopidae) are xylem-feeding insects that are best known for the spittle mass produced by their nymphs [4,5]. Spittlebug nymphs surround themselves with a frothy, spittle-like foam, which is produced by foaming up an excreted protein-rich liquid [6]. The nymphs of several spittlebug species are known to feed gregariously [3,5,7-10]. *Callitettix versicolor* (Fabr.) is a serious pest in both rice, maize, and several other economically important crops in China and SE-Asia [11]. During studying the biology of this pest, we found that the nymphs of this species feed gregariously like other spittlebugs. As shown in Figure 1, there are usually several nymphs of *C. versicolor* in one spittle mass.

Despite their limited motility, young spittlebug nymphs move from the hatching spot to choose appropriate feeding sites [10]. Several spittlebug nymphs usually share one spittle mass on their host plant, even if many other apparently suitable feeding sites on the plant remain unoccupied [5]. Hence, spittlebug aggregation during immature stages is not the result of clustered eggs or limited host plant resources. By contrast, this kind of aggregation behavior appears to be the result of active crowding, for which as yet unidentified aggregation pheromones are likely to be important. Because the nymphs of spittlebug are covered with spittle mass, some chemical components of the spittle mass may act as aggregation pheromones. We therefore proposed that aggregation behavior of these nymphs is associated with specific components of the spittle mass. However, most of the previous investigations focused on the macromolecular components of the spittle mass and the difference in composition between the spittle mass and the insect’s diet [12-14]. Little work has been undertaken so far to analyze the presence of volatile organic compounds (VOCs) emitted by the spittle mass, as well as their function during the aggregation process. The purpose of this study was to investigate whether the nymphs of *C. versicolor* use particular VOCs in the spittle mass to regulate their aggregation behavior.

## Results

### Aggregation behavior

**Figure 2:**
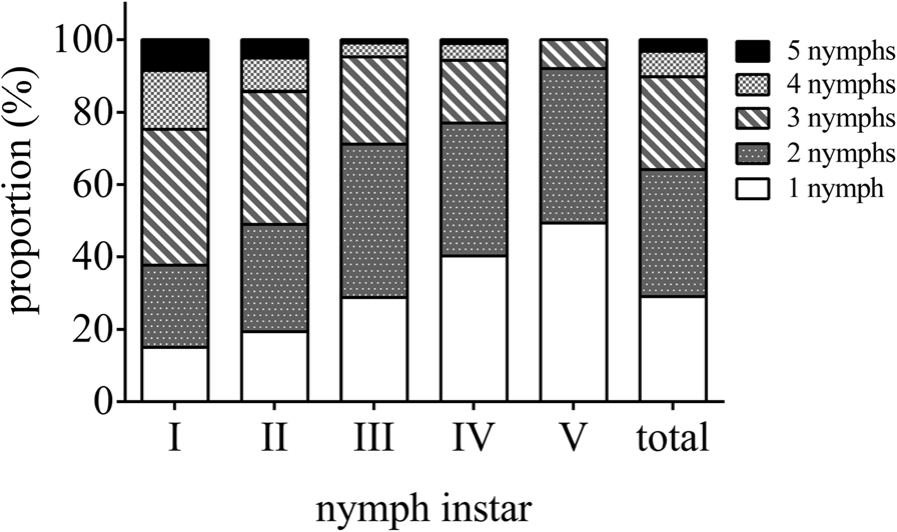
Aggregation behavior of the nymphs of *C. versicolor* in field.

In order to investigate the aggregation behavior of nymphs, we performed field assays using a quadrat sampling method [3]. Spittle masses with only one nymph was defined as solitary spittle mass (abbreviated as SSM), while spittle masses with more than one nymphs were defined as aggregation spittle masses (abbreviated as ASM). As shown in Figure 2, the nymphs of *C. versicolor* aggregated in spittle masses, with up to 5 nymphs in every individual spittle mass. In the two patches studied, over 60% of spittle masses contained 2 or 3 nymphs. In general, the degree of aggregation decreased with successive instars. The highest aggregation levels occurred in instar I, where 84.9% lived in aggregations. Nymphs of the fifth instar stage were never found to aggregate beyond threesomes in both patches. The degree of aggregation of all nymphs in these two patches were similar. Thus, in the field, most nymphs of *C. versicolor* were found to live in aggregation.

### Chemical identification and quantification

**Figure 3:**
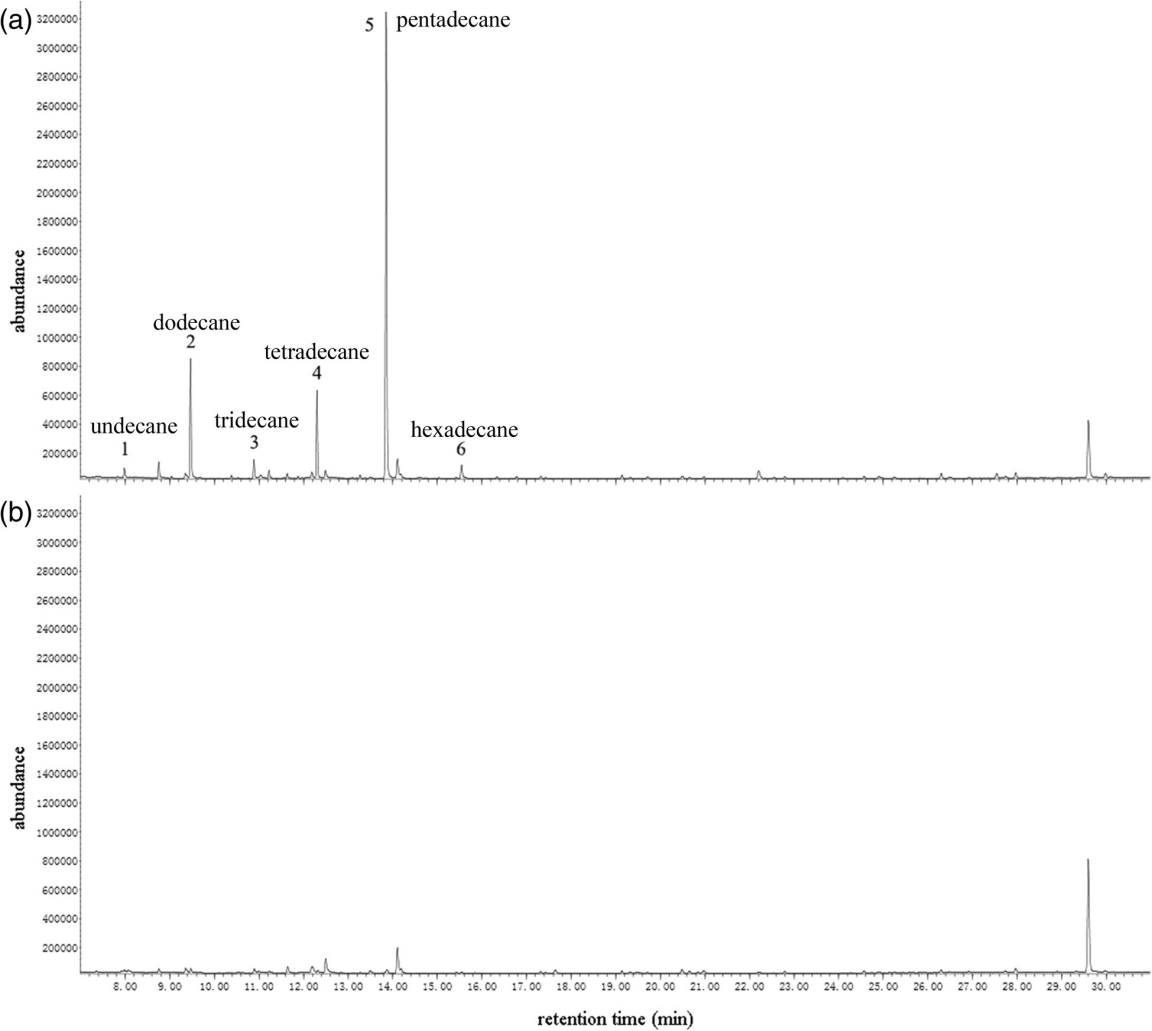
Total ion chromatograms of the hexane extractions of **(a)** spittle masses and **(b)** rice seedlings root.

In order to analyze the volatile components of the spittle mass, we performed chemical identifications using GC-MS (gas chromatography–mass spectrometry) analysis. Previous studies have shown that some macromolecular components (e.g. polypeptide, proteins, and polysaccharide) of the spittle mass may have derived from exogenous sources, including host plant juices [14]. In this study, extracts of rice-seedling roots were used as control, to determine whether the chemical components of spittle mass were from the host plant juice or secreted by the nymphs. For chemical identification, spittle masses were randomly selected without separating aggregation or solitary types. As shown in Figure 3, we identified six unique compounds in the GC-MS chromatograms obtained from spittle mass extracts, all of which were absent in the rice-seedling root extracts. These compounds were identified as a series of *n*-alkanes: undecane (C11), dodecane (C12), tridecane (C13), tetradecane (C14), pentadecane (C15) and hexadecane (C16). The absolute amounts of these six compounds per spittle mass were calculated for the different spittle types according to the calibration regression equations.

**Figure 4:**
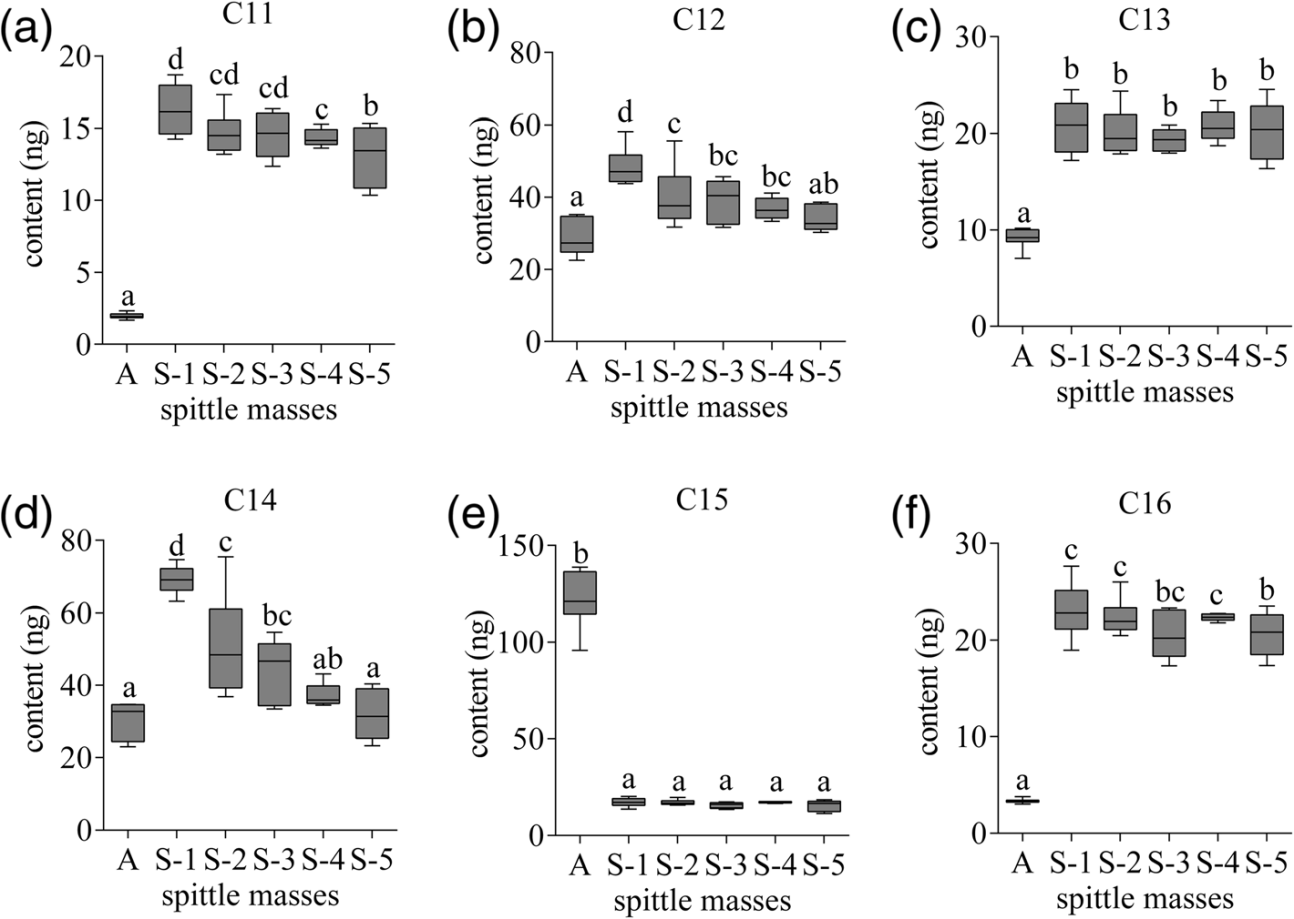
The contents (*N* = 10–15 spittle masses, 2 g each) of **(a)** undecane, **(b)** dodecane, **(c)** tridecane, **(d)** tetradecane, **(e)** pentadecane and **(f)** hexadecane in different types of spittle masses. There were significant differences between the contents with different characters (*p* < 0.05, one-way ANOVA with LSD test). A: aggregation spittle mass produced by different instars nymphs (the nymphs in one spittle mass may be not at the same instar); S-1: solitary spittle mass produced by the first instar nymphs; S-2: solitary spittle mass produced by the second instar nymphs; S-3: solitary spittle mass produced by the third instar nymphs; S-4: solitary spittle mass produced by the fourth instar nymphs; S-5: solitary spittle mass produced by the fifth instar nymphs.

The amounts of these compounds differed significantly between SSM and ASM (Figure 4). The amounts of C11, C12, C13, C14 and C16 in SSM produced by nymphs of each instar were all higher than those in ASM. The amounts of C11, C12, C14 and C16 in SSM generally decreased with successive instars. Furthermore, no significant differences were found in the amounts of C13 and C15 in SSM among different instars.

Next, we analyzed the composition of specific *n*-alkanes in ASM versus SSM. The amounts of C11, C13 and C16 produced by all instar nymphs were significantly higher in SSM than that in ASM. Although the amounts of C12 and C14 in SSM produced by the fourth or fifth instar nymphs were not higher than those in ASM, their levels were significantly higher in the first, second and third instar nymphs of SSM relative to ASM. In contrast, the amounts of C15 in ASM were significantly higher than those in SSM produced by all instars nymphs. Together, our results suggest that the levels of specific *n*-alkanes correlate with the state of spittle mass, and that their amounts are regulated by the nymphs according to the aggregation state.

### EAG responses

**Figure 5:**
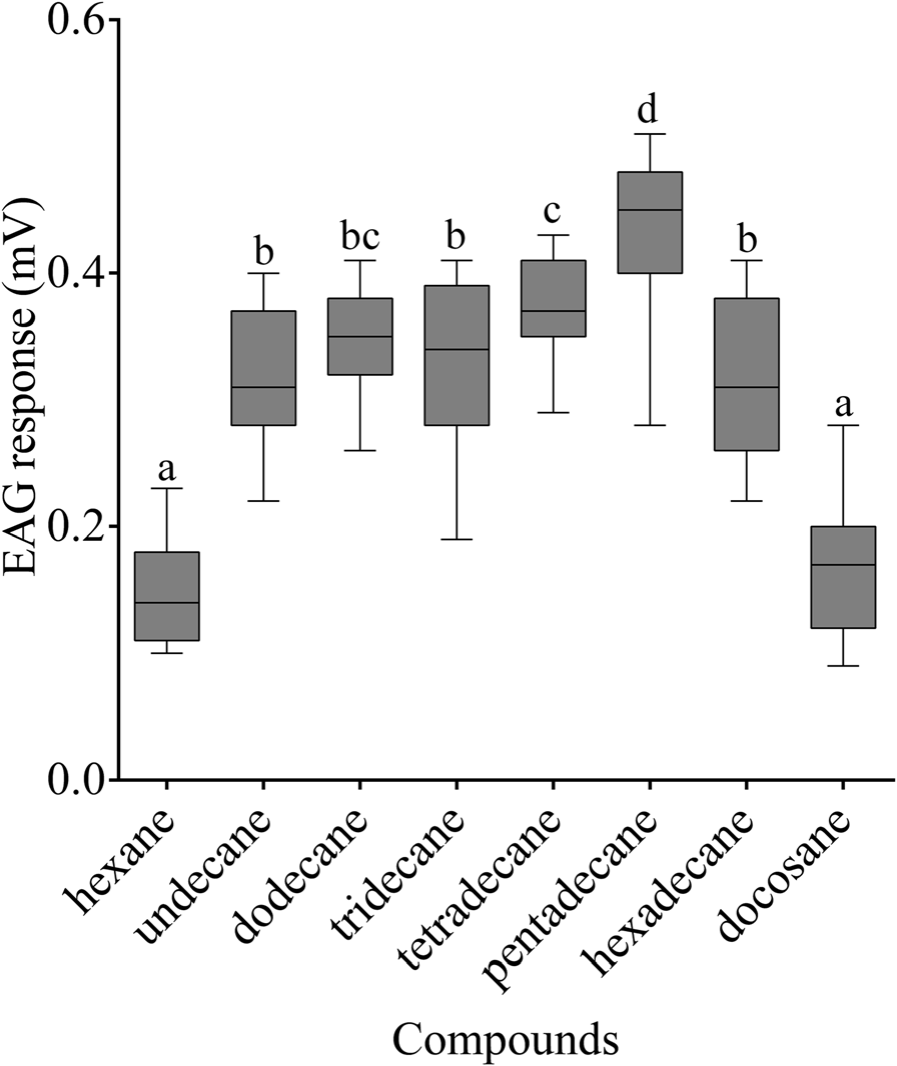
EAG responses (*N* = 15) of *C. versicolor* nymphs to the synthetic compounds. There were significant differences between nymph responses with different characters (*p* < 0.05, one-way ANOVA with LSD test).

In order to verify whether the nymphs display an electrophysiological response to these six compounds, Electroantennogram (EAG) recordings were prepared. All six synthetic compounds released EAG responses in the antennae of nymphs (Figure 5). The antennae gave the highest response to C15. The EAG responses to C11, C12, C13, C14 and C16 were also significantly higher than the response to hexane and the docosane (negative controls). Together, these results indicated that all six compounds are potential components of a pheromone system that regulates aggregation behavior.

### Behavioral responses

#### (1) Behavioral response of nymphs towards established SSM and ASM

**Figure 6:**
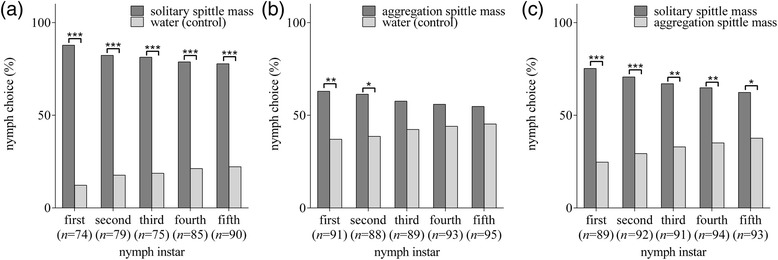
Behavioral responses of different instar nymphs of *C. versicolor* to solitary spittle masses and aggregation spittle masses (*Indicates *p* < 0.05, **Indicates *p* < 0.01, ***Indicates *p* < 0.001, Chi-square test). **(a)** solitary spittle mass vs. water (control); **(b)** aggregation spittle vs. water (control); **(c)** solitary spittle mass vs. aggregation spittle mass.

To investigate behavioral responses of nymphs to the two types of spittle masses, the attractiveness of ASM and SSM to nymphs was tested. In the SSM assay, nymphs in different instars all displayed significant preference to spittle mass compared to the water control (Figure 6a). These results indicated that all instar nymphs can be attracted by SSM, and suggest that aggregation behavior is possibly regulated by spittle mass.

Next, we tested the behavioral responses of nymphs to established ASM. Nymphs in the first and second instars displayed significant preference to ASM compared to the water control (Figure 6b). However, nymphs in the third, fourth and fifth instars had no significant preference to ASM compared to the control.

Lastly, we compared the choice rates of nymphs to SSM with those to ASM (Figure 6c). The choice rates of all instar nymphs to ASM were significantly lower than those to SSM. These results indicated that SSM were preferred by all instar nymphs over ASM.

#### (2) Behavioral responses of nymphs to single compounds

**Figure 7:**
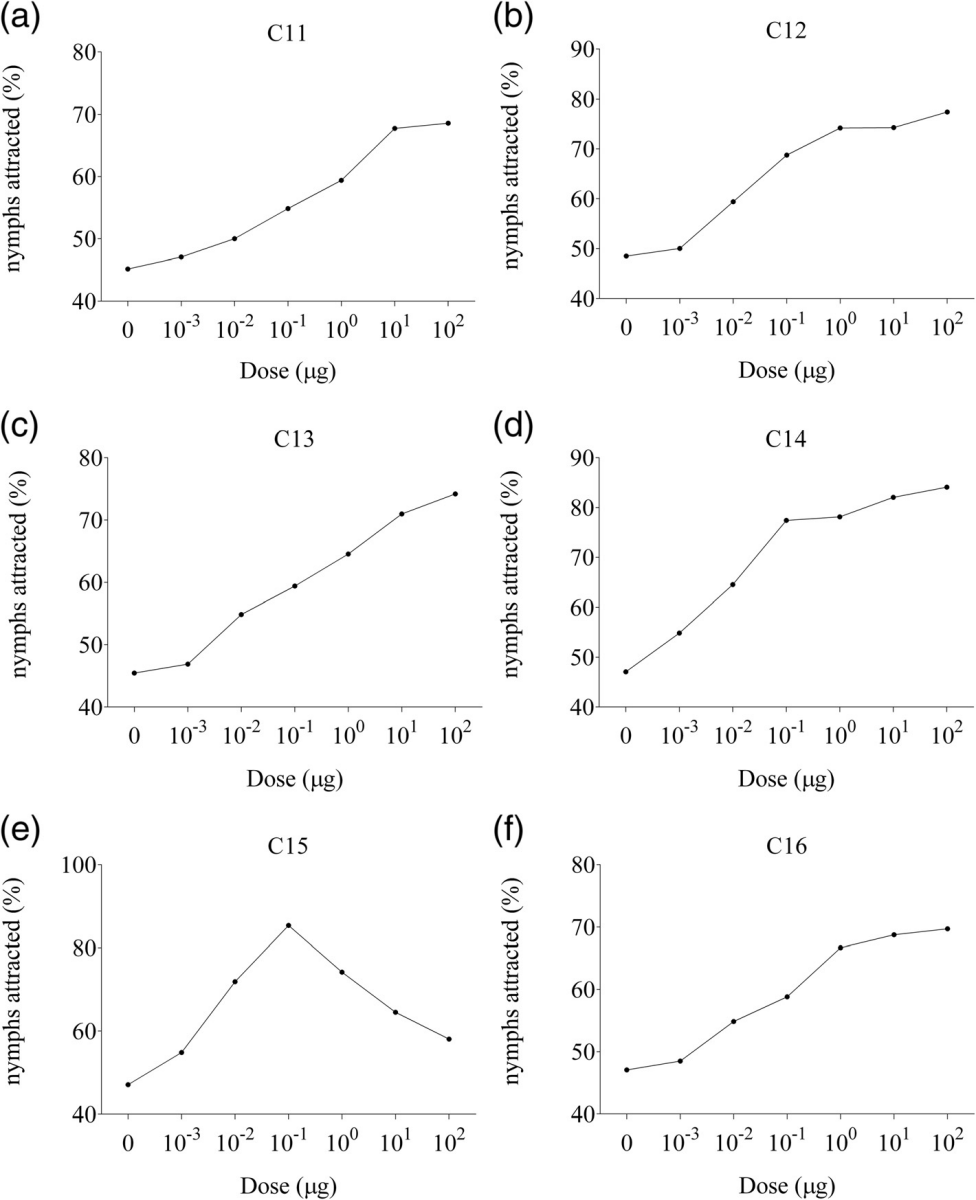
Behavioral responses of fifth instar nymphs of *C. versicolor* to various doses of **(a)** undecane, **(b)** dodecane, **(c)** tridecane, **(d)** tetradecane, **(e)** pentadecane and **(f)** hexadecane. For every compound, and in each assay, 30 to 50 nymphs were tested.

In order to verify the behavioral response of nymphs to each compound, we performed behavioral tests with individual alkanes. The behavioral response of nymphs to C11 (Figure 7a), C12 (Figure 7b), C13 (Figure 7c), C14 (Figure 7d) and C16 (Figure 7f) showed a positive correlation with the doses tested. However, the response to C15 (Figure 7e) was different from the responses to the other five compounds. C15 exposure resulted in an increase in choice rates at lower doses ranges (0.001, 0.01, and 0.1 μg). In contrast, the choice rates declined at higher dosages (1, 10, and 100 μg). The critical dose (100 ng) of C15 in the behavioral assay corresponded with its natural dose (120.83 ± 5.50 ng) in ASM. These results indicated that the attractiveness of C15 to nymphs declined at higher doses, and suggested that the C15 plays a role that is different to that of the other five compounds.

#### (3) Behavioral responses of nymphs to specific compound mixtures

**Figure 8:**
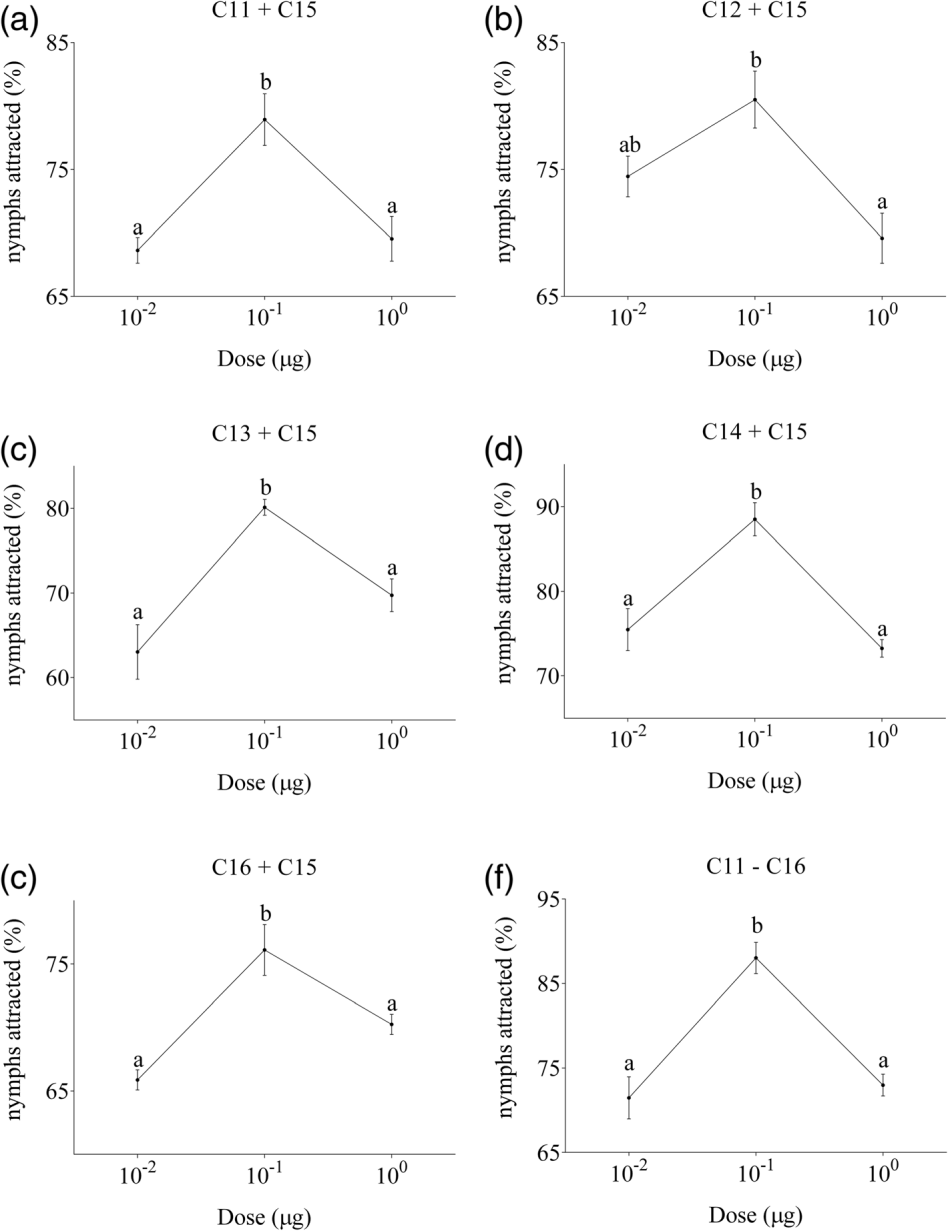
Behavioral responses of fifth instar nymphs of *C. versicolor* to various doses of mixtures of **(a)** undecane + pentadecane, **(b)** dodecane + pentadecane, **(c)** tridecane + pentadecane, **(d)** tetradecane + pentadecane, **(e)** hexadecane + pentadecane and **(f)** all these six compounds. Each assay block consisted of testing 10 to 20 nymphs individually and mean ± SE were calculated from 3 blocks of assays. There were significant differences between the responses of nymphs with different characters (*p* < 0.05, one-way ANOVA with LSD test).

In order to analyze the interaction of C15 with the other five compounds, we tested the behavioral responses to specific mixtures of compounds. C15 (0.01, 0.1 and 1 μg) was mixed with equal amounts of either C11, C12, C13, C14, C16 and a combination of ALL. As shown in Figure 8, we observed significant differences in the responses of the nymphs to these mixtures. Similar to C15 alone, choice rates increased from 0.01 to 0.1 μg C15 per simple, but decreased from 0.1 to 1 μg C15 per simple, indicating that the five other alkanes do not interfere with the function of C15 in attracting nymphs.

## Discussion

The results of our study presented here indicate that the aggregation behavior of spittlebug nymphs is not a random, but a highly regulated process. Our analysis revealed that this behavior is induced and regulated by aggregation pheromones in the spittle mass. Previous reports describing the analysis of spittle masses, from various spittlebug species, suggested that spittle mass is a complex matrix that contains multiple classes of compounds, including proteins, polysaccharides and cellular metabolites [12-14]. However, previous studies only focused on the composition of spittle mass, yet neglected its function in regulating nymph behavior. Furthermore, small molecules and volatile components, such as the *n*-alkanes identified here, had not been identified in spittle masses of spittlebugs previously. In the present study, we revealed that highly specific chemical compounds in the spittle mass play an important role in regulating the aggregation behavior of spittlebug nymphs. To our knowledge, this is the first identification of aggregation pheromones in spittle mass that regulate aggregation behavior of spittlebug nymphs. In view of aggregation pheromones having been widely explored in pest management, this study offers new options for the control and forecast of this pest by using pheromones.

Pheromones are usually multicomponent mixtures of compounds [15]. Although a number of species may share some particular components, each species appears to produce its own particular mixture [16]. In this study, a homologous series of *n*-alkanes were identified, and revealed to act as aggregation pheromones of *C. versicolor* nymphs. Although *n*-alkanes are simple in chemical terms, the ways in which insects and other arthropods have evolved to use them as semiochemicals are far from simple [15]. Semiochemical functions attributed to *n*-alkanes include, for example, sex pheromones and aphrodisiacs [17,18], species and caste recognition cues [19,20], territory marking [21], recruitment and alarm pheromones [22,23], as well as defensive secretions [15,24]. Some of these six compounds identified in the spittle mass produced by *C. versicolor* nymphs also act as aggregation pheromones in a number of Hemiptera species. For example, tridecane is the major component of thee aggregation pheromones of six stink bugs species [25]; pentadecane is the component of aggregation pheromones of house dust mites *Dermatophagoides pteronyssinus* [26]. In addition, it has been reported that nymphs of four different spittlebug species coexisted in the same spittle mass [27], suggesting that nymphs of these species share the same aggregation pheromones. Pheromones in Hemiptera are usually produced in specialized scent glands, which are localized in the abdomen in immature or in the metathorax in adults and some compounds are extensively shared across species, genera and even families [16]. Therefore it is likely that the aggregation pheromones of *C. versicolor* nymphs may be first secreted by specialized scent glands and then mixed with the spittle mass, instead of being secreted together with the spittle mass and produced in the malpighian tubules. Furthermore, in insects, these *n*-alkanes are common components of cuticular hydrocarbons, and the major site of hydrocarbon biosynthesis occurs in the oenocytes associated with the epidermal layer or peripheral fat body [28]. Hence, these compounds may also be secreted from the epidermal layer or the fat body, to be further mixed and dissolved in the spittle mass. It will be important to investigate the origin of these compound in future investigations.

In nature, each trait of an organism is shaped throughout the long process of evolution, and is often based on a balance between costs and benefits, as observed for the aggregation behavior of nonsocial animals [1]. Such aggregation of nymphs of spittlebugs may have advantages for exploiting food resources [3]. Also, gregarious feeding might assist in overcoming barriers presented by the host plant [5]. Despite the spittlebug nymphs (and other xylem feeders) have anatomical adaptations to overcome the enormous negative pressures inside the xylem [29], multiple nymphs simultaneous sucking xylem may help nymphs, particularly small, early instar nymphs, to break this tension more effectively [5]. Moreover, it was shown that aggregated nymphs are able to re-build the protecting spittle mass more quickly than single nymphs following destruction of the spittle mass [3]. Furthermore, a recent report indicated that each nymph in an aggregation can reduce its contribution to the production of spittle mass, thus saving energy and resources [7]. Nymphs need to excrete copious amounts of fluid (up to 284 times their body mass per day), which is a time- and energy-consuming process [5]. In agreement with this, it has been shown that production of foams (spittle mass) is energetically expensive and difficult to maintain, because formation of bubbles in liquids requires considerable energy input to overcome the high surface tension and increased surface energy at the exposed gas–liquid interface [30]. Hence, when in aggregation, nymphs can maintain spittle mass more easily than when in solitary. Consequently, the aggregation usually result in reduced mortality of nymphs, and is regarded as an advantageous state for nymphs of spittlebugs [3,8]. However, individuals within aggregations experience higher costs than they would if they were solitary, and competition for food and space may be the most severe challenge [1]. At higher densities the aggregation may be limited by feeding site availability, so that intra-specific competition may increase and override the beneficial effect of aggregation [3]. The increase in nymphal mortality with increasing group size and the decrease in mass at adult eclosion that occurred when in excessive aggregation suggest that nymphs were competing for limited plant resources [5]. Moreover, the content or flow of xylem sap may be deteriorated during longer periods of draught and competition between nymphs in one spittle may occur [3].

Our study suggests that the number of nymphs in each spittle mass is allocated for optimal aggregation size. Likewise, previous studies concerning the behavior and ecology of spittlebug nymphs, showed that the number of nymphs in each spittle mass ranges from one to eight in different spittlebug species [3,5,31]. In this study, we found a maximum of five nymphs per spittle mass. Such aggregations of nonsocial animals are usually induced and regulated by aggregation pheromones, which are produced by the aggregating individuals themselves. After aggregation reaches a certain group size, it is commonly observed that under crowded conditions, production of aggregation pheromones ceases [1]. In addition, high doses of some pheromones have been shown to have repelling effects on potential co-inhabitants. Furthermore, infochemicals are used to prevent further aggregation. For example, *trans*-verbenol, verbenone and ipsdienol, can limit the density of bark beetle, *Dendroctonus brevicomis*, by changing the ratio of pheromones that attract these beetles to their tree habitats [32]. In our study, we revealed that the aggregation behavior of *C. versicolor* nymphs is induced by aggregation pheromones within the spittle mass. Furthermore, the aggregation pheromones are not static in terms of quantities and qualities, and they are not only just used as cues for nymphs to aggregate; the pheromones are actively produced and the amount and ratios of them in spittle mass are dynamically regulated by nymphs to maintain the aggregation at optimal size. Our study suggests that the nymphs can regulate the levels of these pheromones according to the aggregation status of the spittle mass. The nymphs will increase amounts of C11, C12, C13, C14 and C16, and decrease amounts of C15 in their spittle mass, when they are solitary; whereas, they will increase amounts of C15, and decrease amounts of C11, C12, C13, C14 and C16, when they are in aggregation. Moreover, C15 attracts nymphs at lower doses in SSM, and this attractiveness declines once present at higher doses in ASM. The dose-dependent role of C15 is likely to allow for more efficient regulation of the aggregation process. Thus, our study reveals that the spittle mass does not only contain the metabolites of nymphs as reported previously. As supported by the strong evidence in the present study, the spittle mass is an efficient yet simple self-regulatory system that both affects the aggregation behavior of nymphs as well as balances the benefits and costs of aggregation.

The features of pheromone-regulated aggregation behavior of spittlebug nymphs reported here show aspects of convergent evolution for the optimizing of aggregation of nonsocial animals, and is instructive for studying the aggregation of other animals. Moreover, our findings broaden our understanding of aggregation behavior of nonsocial animals, and shed light on exploring the pheromone-regulated aggregation of nonsocial animals from an evolutionary and ecological perspective.

## Conclusions

In this study, we found that *C. versicolor* nymphs of all instar stages are attracted to both SSM and ASM, with a significant preference for the former. Together with the results from our behavioral essays and biochemical analysis, this clearly demonstrates that aggregation behavior is induced and regulated by the spittle mass itself, and that its volatile components play important roles in the aggregation process. Although the volatile components, which were identified as C11, C12, C13, C14 and C16 in this study, are not the main compositions of spittle mass and had been neglected before, they appear essential for chemical communications of nymphs and their amounts are precisely regulated. Furthermore, these pheromones were found to play different roles in regulating the aggregation behavior of nymphs. The C11, C12, C13, C14 and C16 acted as attractants to nymphs at all doses, and can thus be seen as primary recruitment agents that attract nymphs to join a spittle mass, resulting in aggregation. For C15, however, the role as an attractant diminished with increasing doses. Present in low doses in SSM, its function as a nymph attractant is aligned with that of the other alkanes. In ASM, it is present in higher doses, which turns this alkane into a suppressive factor that overrides the weak attraction of the other five compounds present in low doses, thus, preventing any further aggregation and over-crowding. In conclusion, we describe here an excellent example of biological self-regulation, whereby a limited number of simple chemical compounds exert their forces onto a complex behavior, thus maximizing the survival chance of individual nymphs within a population of competing individuals.

## Material and methods

### Insect

The nymphs of *C. versicolor* were reared as reported earlier [33] with minor modifications, spittle mass was collected, and behavioral assays were performed as described below. The nymphs were reared on rice seedlings in plastic boxes (50 cm in length, 40 cm in width, and 20 cm in height), with 10–20 nymphs in each box. Nymphs were maintained inside an artificial climate chamber at 27 ± 1°C, a photoperiod of 14:10 (L: D) h, and 70 ± 5% relative humidity. The bottom (5–10 cm) of each box was surrounded with a black cloth, to provide a darkened environment for nymphs, to mimic their natural conditions.

### Field survey

Field experiments were conducted in Guizhou province, China, in August 2014, during a time when the amount of spittle masses was at its maximum. Two large patches with weeds were chosen for studying the aggregation behavior of *C. versicolor* nymphs. One patch was chosen in a vineyard (named P1, approximately 30 m × 40 m) and contained predominantly weed. The second patch (named P2, approximately 20 m × 30 m) was uncultivated land located beside a rice field. The density of nymphs was calculated as 5.7 nymphs per 0.25 m^2^ (n = 10, S.D. = 2.21) in the study area. The aggregation of nymphs was measured using the quadrat sampling method, which is frequently used to quantify the density of spittlebug nymphs [3]. At each of the two patches, spittle masses were examined in plots of 0.5 m × 0.5 m, with 50 plots chosen randomly for analysis. Roots were dug out for examination, as the nymphs live on both roots and stalks near the ground. When present, spittle masses on stalks further up and leaves were also examined. The nymphs in each spittle mass were counted, and their instars were determined according to morphological characteristics [33]. The degree of aggregation was calculated for each instar as the proportion of nymphs that lived in nymphal aggregations [3].

### Chemical procedures

Two grams of spittle mass were collected and injected into a glass amber vial (Agilent Technologies, Santa Clara, California, USA) using a 1 ml glass syringe (Agilent Technologies, Santa Clara, California, USA). In addition, 2 g of rice seedling roots void of nymphs and spittle masses were shredded and transferred into identical vials. Two ml of hexane (solvent) (Sigma-Aldrich, St. Louis, Missouri, USA) was added into each vial containing either spittle mass or rice seedling root samples. The mixtures were shaken for 5 minutes on a shaker platform (MS1 Mini Shaker Vortex, IKA, Germany) at room temperature, and then left to incubate for 12 hours at 4°C. The mixtures were then centrifuged at 10000 rpm for 5 minutes, and the hexane phases were transferred into new vials. The residues in both vials were extracted with 1 ml hexane again, followed by another centrifugation step. The extractions were concentrated to 100 μl using nitrogen (purity great than 99.9%) and stored at −20°C for analysis.

The extractions from both spittle masses and rice seedling roots were analyzed by gas chromatography - mass spectrometry (GC-MS) (Agilent 6890 N GC coupled to 5973 MS). The gas chromatograph was equipped with a HP5-MS capillary column (30 m long, 0.25 mm internal diameter, 25 μm film thickness; Agilent Technologies, Santa Clara, California, USA). Helium was used as a carrier gas, at a flow rate of 1.0 ml min^− 1^. The temperature of the injector was set to 250°C. The temperature of the GC oven was held at 50°C for 1 min, and then increased to 280°C at 5°C per minute, and held for 2 min. The MS transfer line was held at 300°C, the MS source at 230°C, and the MS quad at 150°C. Mass spectra were produced in electron ionization mode (at 70 eV) in the mass range of m/z 30 to 450. GC-MS data were processed with the MSD-ChemStation software (Agilent Technologies, Santa Clara, California, USA). Compounds were provisionally identified by matching their gas chromatographic retention times and mass spectra with authentic analogues within the NIST 2.0 mass spectra database using the NIST algorithm. Identified compounds were then confirmed by comparing retention times and spectra with synthetic standards for C11, C12, C13, C14, C15 and C16 (all were standard material for GC, purity greater than 99.5%, Tokyo Chemical Industry Co., Ltd., Tokyo, Japan).

To quantify the compounds in spittle mass, samples consisting of C11, C12, C13, C14, C15 and C16 at different concentrations (1, 20, 40, 80, 120, 160, or 200 ng/μl) were used as external standards for generating standard curves for quantification. To quantify the compounds in ASM, nymphs were reared as mentioned above. ASM were selected randomly and collected individually. Single spittle masses were transferred into a 250 μl vials (Agilent Technologies, USA) and 100 μl hexane was added. The mixture was then processed as described above. The extracts were concentrated to 10 μl and stored at −20°C until further processing. For quantification of compounds in SSM, after hatching, nymphs were reared individually with rice seedlings in separate grids (7.5 cm × 7.5 cm), to prevent the aggregation inside the boxes (15 grids per box). The plastic boxes were put in the same climate chamber. The spittle masses were collected individually on day 3 of each instar, and then processed as described above. Ten to fifteen spittle masses of each type were quantified.

### Electroantennogram (EAG) recording

Electroantennogram (EAG) recordings were prepared by placing a *C. versicolor* nymph antenna between two silver wires electrodes that were covered by thin glass capillaries filled with Ringers solution. Antennal responses were recorded via an amplifier connected to a signal acquisition interface board (IDAC, Syntech, Hilversum, The Netherlands) for signal transfer to a PC. Antennal preparations were exposed continuously to charcoal-filtered and moistened air at a velocity of 250 ml min^− 1^. The six synthetic compounds and docosane (second negative control used to test whether antennae responses were specific for spittle mass compounds) were dissolved in hexane at 0.1 μg μl^− 1^. Ten microliters of each test compound or pure hexane (control) was absorbed on a piece of filter paper strip (40 mm length, 5 mm width). The filter paper strip was inserted into a clean glass pipette, and the solvent was then allowed to evaporate from the filter paper (~30 s) prior to testing. The test compound was delivered via a 1 s puff of air (5 ml s^− 1^) into an opening in the glass tube 150 mm upstream from the antenna, with the outlet 10 mm downstream from the antenna [34]. Before starting each run, a single pulse of air was blown onto the antenna to verify that it was responsive. Pure hexane was used as control, with treatment order of the test compounds randomized. The EAG responses were tested on the antennae of 15 nymphs at the fifth instar stage.

### Behavioral assays

**Figure 9:**
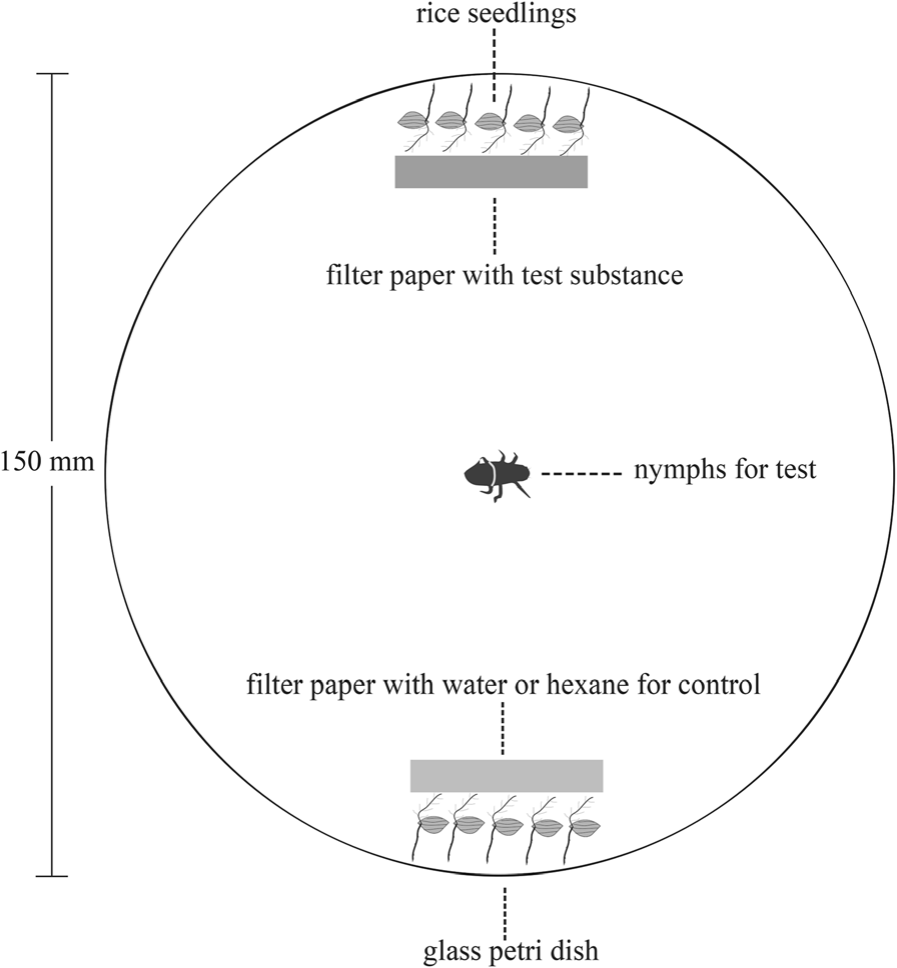
Apparatus for behavioral assays. Five rice seedlings without soil and filter paper with test substance or water/hexane (control) were put at the two opposite sides. The nymphs were individually introduced into the center of a glass Petri dish to test.

Nymphs of *C. versicolor* normally feed on root of rice or weeds underground or near the ground, where it is wet, dark and windless. Therefore, a still air apparatus was used for our behavioral studies. Behavioral assays were conducted in glass Petri dishes (15 cm × 3 cm, diameter × height) (Figure 9). Moistened absorbent cotton covered by a piece of filter paper was placed into a glass Petri dish to provide a moist surface and to simulate a humid environment for nymphs tested (the humidity was 90 ± 5 % inside the Petri dish). During testing, both test substances and the control (water or hexane) were loaded onto filter paper strips (2 cm in length, 1 cm in width) before the filter paper strips were put beside the rice seedlings at the two opposite sides. Five rice seedlings (with soil removed) were placed beside filter papers at each of the two opposite sides, to allow the nymphs to remain at the side of choice. Because the fifth instar is the most resilient of all stages to experimental handling, and also much easier to operate in behavioral assays, these nymphs were used for behavioral assays 1–2 days after molting, unless noted otherwise. Prior to test start, nymphs were individually placed into the Petri dishes without rice seedlings for 30 min. For testing, nymphs were placed individually into the center of the Petri dish, and the Petri dish was then covered with a lid. The test glass Petri dishes were covered with one layer of black cloth during testing, to provide a dark environment for nymphs and to prevent any light interference. The behavioral assays were all conducted at room temperature (25 ± 2°C) and always between 9:00 AM and 12:00 AM. Each nymph was tested only once.

#### (1) Test of spittle mass

To analyze the degree of attractiveness of ASM and SSM to nymphs of each instar, we compared the number of nymphs that chose either of the two masses. Third instar nymphs were fed individually in glass Petri dishes with rice seedlings for SSM collecting. The spittle masses were collected after 48 h. For the collecting of ASM, the nymphs were reared as described above. In the separate test of SSM or ASM, 0.1 g of spittle mass (this quantity is roughly equivalent to that produced by 1 third or fourth instar nymph in 48 h) was put beside the rice seedlings at one side of the test glass Petri dish, and 0.1 g deionized water (control) was put alongside the rice seedlings at the opposite side. In the test of SSM together with ASM, the SSM and ASM were put separately at the two sides of the Petri dish. Nymphs of all instars (the first, second, third, fourth and fifth) were used for the tests. The choices of nymphs were recorded after 5 min. The Petri dish was replaced after every five nymphs tested to avoid the influence of odor left by the previous nymphs. 80–100 nymphs of each instar were tested individually. The nymphs that did not make choices were discarded.

#### (2) Test of single compounds

C11, C12, C13, C14, C15 and C16 were dissolved in hexane at concentrations of 0, 0.0001, 0.001, 0.01, 0.1, 1 or 10 μg μl^− 1^. 10 μl of these test compounds and hexane (solvent control) were loaded onto individual filter paper strips. After the solvent evaporated (~30 s), the filter papers were placed into Petri dishes beside the rice seedlings at opposite sides of the Petri dishes. These six compounds were tested individually. The choices of the nymphs were recorded after 5 min. Nymphs that did not make choices were discarded. For every compound, and in each assay, 30 to 50 nymphs were tested.

#### (3) Test of mixtures of compounds

Mixtures of C15 with the other five compounds (C11 + C15; C12 + C15; C13 + C15; C14 + C15; C16 + C15), and one mix of all six compounds (C11 + C12 + C13 + C14 + C15 + C16) were tested to examine the effect of these mixtures on nymph behavior. These mixes were all prepared in hexane at concentrations for all compounds at 0.001, 0.01or 0.1 μg μl^− 1^ (the concentrations were selected based on the results of the single compound tests, which covered low, moderate and high concentration ranges). The tests were completed as described above.

### Statistical analysis

For quantitative assays, differences in amounts of each compound in the different samples were analyzed by one-way analysis of variance (ANOVA) with the least significant difference (LSD) test.

For the electrophysiology assays, differences in EAG responses for different odor stimuli were analyzed by one-way analysis of variance (ANOVA) with LSD test.

In the spittle mass behavioral assays, the Chi-square test was used to determine whether spittle masses were attractive to the test nymphs.

In the behavioral assays using mixtures of synthetic compounds, differences in nymphs’ response to various doses were analyzed by ANOVA with LSD test. In the single compound test, the nymphs’ responses to various doses were analyzed using linear regression analysis of log (x + 1) transformed data to confirm the linear relationship between them.

All statistical analyses were conducted using SPSS Statistics Version 19.0 (Windows 7).
